# Setting health priorities in a community: a case example

**DOI:** 10.1590/S1518-8787.2017051006460

**Published:** 2017-02-23

**Authors:** Fábio Alexandre Melo do Rego Sousa, Maria José Garcia Goulart, Antonieta Manuela dos Santos Braga, Clara Maria Oliveira Medeiros, Débora Cristina Martins Rego, Flávio Garcia Vieira, Helder José Alves da Rocha Pereira, Helena Margarida Correia Vicente Tavares, Marta Maria Puim Loura

**Affiliations:** I Centro de Saúde de Ponta Delgada. Unidade de Saúde de Ilha de São Miguel. Ponta Delgada, Açores, Portugal; II Centro de Saúde de Vila do Porto. Unidade de Saúde de Ilha de Santa Maria. Vila do Porto, Açores, Portugal; III Unidade de Saúde Pública. Unidade de Saúde de Ilha de São Miguel. Ponta Delgada, Açores, Portugal; IV Departamento de Enfermagem, Saúde da Família e Comunidade. Escola Superior de Saúde. Universidade dos Açores. Ponta Delgada, Açores, Portugal; V Serviço de Hemodiálise. Hospital do Divino Espírito Santo, EPE. Ponta Delgada, Açores, Portugal; VI Serviço de Saúde Ocupacional. Hospital do Divino Espírito Santo, EPE. Ponta Delgada, Açores, Portugal

**Keywords:** Health Priorities, Health Planning, methods, Planning Techniques, Social Planning, Consumer Participation, Health Inequalities, Hanlon Method

## Abstract

**OBJECTIVE:**

To describe the methodology used in the process of setting health priorities for community intervention in a community of older adults.

**METHODS:**

Based on the results of a health diagnosis related to active aging, a prioritization process was conceived to select the priority intervention problem. The process comprised four successive phases of problem analysis and classification: (1) grouping by level of similarity, (2) classification according to epidemiological criteria, (3) ordering by experts, and (4) application of the Hanlon method. These stages combined, in an integrated manner, the views of health team professionals, community nursing and gerontology experts, and the actual community.

**RESULTS:**

The first stage grouped the identified problems by level of similarity, comprising a body of 19 issues for analysis. In the second stage these problems were classified by the health team members by epidemiological criteria (size, vulnerability, and transcendence). The nine most relevant problems resulting from the second stage of the process were submitted to expert analysis and the five most pertinent problems were selected. The last step identified the priority issue for intervention in this specific community with the participation of formal and informal community leaders: Low Social Interaction in Community Participation.

**CONCLUSIONS:**

The prioritization process is a key step in health planning, enabling the identification of priority problems to intervene in a given community at a given time. There are no default formulas for selecting priority issues. It is up to each community intervention team to define its own process with different methods/techniques that allow the identification of and intervention in needs classified as priority by the community.

## RESUMO

**OBJETIVO:**

Descrever a metodologia utilizada no processo de estabelecimento de prioridades em saúde para intervenção comunitária, numa comunidade idosa.

**MÉTODOS:**

Partindo dos resultados de um diagnóstico de saúde no âmbito da promoção do envelhecimento ativo, concebeu-se um processo de estabelecimento de prioridades a fim de selecionar o problema prioritário para intervenção. O processo integrou quatro etapas sucessivas de análise e classificação dos problemas: (1) agrupamento por nível de similitude, (2) classificação de acordo com critérios epidemiológicos, (3) ordenação por peritos e (4) aplicação do método de Hanlon. No decurso destas etapas, combinaram-se, de forma integrada, as perspetivas dos profissionais da equipe de saúde, de peritos em enfermagem comunitária e gerontologia e da própria comunidade.

**RESULTADOS:**

Na primeira etapa, agruparam-se por nível de similitude os problemas identificados, constituindo-se um corpo de 19 problemas para análise. Na segunda, esses problemas foram classificados pelos elementos da equipe de saúde, mediante a aplicação de critérios de cariz epidemiológico (magnitude, vulnerabilidade e transcendência). Os nove problemas mais relevantes resultantes da operacionalização da segunda etapa do processo foram submetidos a análise por peritos, e selecionados os cinco problemas com maior pertinência de atuação. Na última etapa, com recurso à participação de líderes formais e informais da comunidade, identificou-se o problema prioritário para intervenção nessa comunidade específica: a Baixa Interação Social na Participação Comunitária.

**CONCLUSÕES:**

O processo de estabelecimento de prioridades é uma etapa fundamental do planejamento em saúde, permitindo identificar os problemas prioritários a intervir numa determinada comunidade e num determinado momento. Não existem fórmulas predeterminadas para a seleção de problemas prioritários. Cabe a cada equipe de intervenção comunitária a definição de um processo próprio com diferentes métodos/técnicas que possibilitem a identificação e intervenção em necessidades classificadas como prioritárias pela comunidade.

## INTRODUCTION

Health intervention implies considering multicausal and multifactorial realities. Community health professionals face the following challenges: maintaining the quality of health interventions in highly complex contexts; using professional practices based on available scientific evidence; and enabling access to effective and proximity health care to promote community empowerment^7^.

Defining priorities in research and developing public health policies reinforce the importance of planning health strategies. Such planning is a process that establishes priorities with goals and continuity, considering the limits set by existing resources and political, social, cultural, institutional, financial-economic, and international backgrounds^12^. These backgrounds are interrelated and mutually influencing, reacting to potential changes. Thus, health planning is a valuable tool for structured intervention in communities and the necessary definition of priorities^8^.

The issues requiring priority intervention in a specific period of time should be contextually defined, since there are seldom enough resources for interventions addressed to all the needs of a given community^5,9^. Thus, methodologies for setting health priorities that scientifically support the decision-making process and guide community intervention are essential.

Different methods/techniques can be used in this process, individually or combined^8,10^. It is up to community intervention teams to define their own staged processes, incorporating criteria and procedures that allow them to identify and intervene in the community’s priority needs.

The objective of this study was to describe the process of setting health priorities for community intervention in a community of older adults.

## METHODS

The process of setting health priorities involved the participation of several actors (health team, experts and community) focused on the promotion of active aging. The problems presented serve to illustrate and describe the way in which an increasingly refined and integrated analysis strategy was conceived to identify the most relevant health problems in a given community in the active perspective of the actors involved. To better understand the problems, it is important to clarify the framework used to interpret active aging. Active aging can be viewed individually and in population groups. Its implementation allows people to realize their potential for physical, social, mental, and welfare development, to socialize according to their needs, desires and capabilities, and to receive adequate protection, security and care when they need help^11,14,16^.

The initial data used in the analysis process stem from a health diagnosis previously performed by the team with 100 independent older adults, aged 65-84, living in the district of Fajã de Baixo, on the island of São Miguel, in the Azores archipelago, Portugal. The diagnosis instrument was a questionnaire based on determinants considered relevant to active aging described in the literature and organized into different domains: material (physical and economic environment), biological (health and biological characteristics), behavioral (lifestyle and adherence to health care), and psychosocial (emotional state, social support network, education and spirituality) determinants^1,9,13,16,a^.

The identification of the health needs of the community of older adults, resulting from the evaluation of those health determinants, allowed the identification of a diverse set of problems. A classification was developed to enable the selection of the priority problem(s) for intervention.

The prioritization phase was structured into four stages, using diversified procedures and criteria:

1. Grouping of health needs according to their level of similarity;
2. Classification of the grouped needs by the community intervention team according to classic epidemiological criteria – magnitude, vulnerability and transcendence^4,8^. Different strategies were used to reduce subjectivity in applying the criteria: a) review of the initial literature/collection of evidence about the grouped problems; b) division of the team into subgroups for independent classification of the list of problems, and c) group discussion and agreement of the classifications assigned to each problem – obtaining consensus.
3. Ordering of the highest scoring problems in the previous stage by experts in gerontology and community nursing and public health. The average value was calculated from the sum of the values corresponding to the position on the priority ranking, determined by each expert, divided by the number of experts;
4. Application of the Hanlon method^2,8,b^ to prioritize the highest ranked problems according to the experts. This method enables the assessment of health needs by applying criteria, aggregated into components, using the formula:

priority order = (A+B) C × D,

where *A* represents the magnitude of the problem; *B* the seriousness of the problem; *C* the effectiveness of the solution; and *D* the feasibility of intervention classified by the acronym PEARL (pertinence, economic feasibility, acceptability, resources, and legality) (Table 1).

**Table 1:** Hanlon Method Components.

Component	Description	Rating
A	Magnitude – extent of the problem for the population.	Numerical from 0 to 10 (based on % of population affected by the problem).
B	Seriousness – is the problem considered serious?	Numerical from 0 to 10
C	Effectiveness – can the problem be easily solved?	Numerical from 0.5 to 1.5 (where 0.5 corresponds to a problem of difficult solution and 1.5 to a problem of easy solution).
D Feasibility		
P	Pertinence – Is it relevant to intervene in the problem, is the intervention appropriate?	0 or 1 (where 0 corresponds to unfeasible and 1 to feasible) Attributing 0 to any one of the (PEARL) dimensions renders the approach to the problem unfeasible.
E	Economic feasibility – Is there economic feasibility for the intervention?	
A	Acceptability – Does the community accept/want an intervention in the problem?	
R	Resources (availability) – Are there resources available for the intervention?	
L	Legality – Does the law allow the intervention?	

Adapted from: The Illinois Project for Local Assessment of Needs. Available from: http://iphionline.org/pdf/IPHI_IPLAN_Workbook_January_2007.pdf

The PEARL component involves discussing community needs with a focus group comprised of community leaders and formal and informal representatives. The focus group included: chairman, priest, two parish social workers, head nurse of the local health center, a local pharmacy official, and four older adults residing in the parish. The participants were selected for performing functions with decision-making power and mobilizing capacity in the parish, as well as their thorough knowledge of the community and its dynamics. Each of the five problems proposed for debate was analyzed considering the following parameters: (i) pertinence of the problem; (ii) level of community acceptance for intervention in the problem; (iii) economic feasibility, and (iv) availability of community resources.

## RESULTS

In step 1, the findings obtained from the community health diagnosis were analyzed according to the different health determinants. This allowed the identification of 37 real and/or potential problems that could negatively influence the aging process. Grouping according to level of similarity led to reclassification into 19 problems (Table 2).

**Table 2:** Problem grouping by level of similarity. Fajã de Baixo, Portugal, 2012.

Problems/Needs detected
1 – Housing conditions conducive to accidents:
•33.0% of older adults have a bathroom far from the bedroom; 63.0% do not have safety devices in the bathroom; 67.0% use stairs to access their house; 57.0% use stairs daily inside the house; in 20.0% of the cases the stairs had no handrail. 42.0% have slippery floors at home; 32.0% have no carpet anti-slip grip; 84.0% have heaters inside the house.
2 – Low social interaction:
•57.0% of older adults prefer staying home to going out and socializing; individual recreational activities are predominant, and for 75.0% the most frequent is watching TV/listening to music.
3 – Feeling of loneliness:
•23.0% Report feeling alone.
4 – Decrease in the level of self-esteem and perception of happiness:
•32.0% report not feeling happy most of the time.
•There is a trend for self-esteem to diminish with age.
5 – Lack of motivation to engage in intervention/social support activities:
•84.0% do not volunteer work and of those, 77.0% do not wish to.
•26.1% do not know the purposes/projects of the parish’s institutions.
6 – Lack of motivation to engage in activities for personal development and future projects:
•84.0% do not volunteer and of those, 77.0% do not wish to.
•88.0% do not study nor want to.
•34.0% report having no life project.
7 – Feeling insecure:
•22.0% of older adults do not feel safe in their area of residence.
8 – Inadequate attitude towards being a potential victim of violence.
•12.1% would not tell anyone if they were a victim of violence.
9 – Lack of regular physical activity:
•57.6% do not do any physical activity.
10 – Inadequate eating habits:
•53.0% of older adults have an unbalanced diet at dinner.
•46.0% of older adults have only 3 meals a day.
•75.0% have a low water intake.
11 – Cohabitation with smokers:
•23.6% of older adults live with people who smoke.
12 – Lack of family doctor:
•45.0% of older adults do not have a family doctor.
13 – Non-adherence to medicine regimen:
•50.6% of older adults forget to take routine medication.
14 – Non-adherence to officially recommended influenza vaccination schedule:
•68.4% did not take the flu vaccine for the period.
15 – Lack of oral health care:
•96.0% of older adults have lost teeth.
•67.3% do not go to the dentist.
16 – Decreased self-perceived visual and auditory acuity:
•45.0% of older adults have vision problems.
•29.0% have hearing problems.
17 – Self-perceived overweight condition:
•46.0% reported being overweight.
18 – Existence of diagnosed chronic diseases:
•92.0% of older adults present chronic pathology (48.0% rheumatic disease, 47.0% cerebrovascular disease, 33.0% diabetes).
19 – Occurrence of falls and fear of falling:
•24.0% of older adults reported having suffered a fall in the previous semester.

The screening of problems in step 2 was carried out by applying three classic epidemiological criteria – magnitude, transcendence and vulnerability^4,8^ – to each of the 19 problems detected by the intervention team members. A symbols scale was used to classify the impact of each problem. The symbol (+) corresponded to low impact (25.0%); the symbol (++) to medium impact (26.0% to 50.0%); the symbol (+++) to high impact (51.0% to 75.0%); and the symbol (++++) to the greatest impact (> 75.0%). The total score was obtained from the sum of the symbols assigned to each one of the three criteria (Table 3).

**Table 3:** Team ranking of grouped needs/problems. Fajã de Baixo, Portugal, 2012.

Highest scoring problems		Lowest scoring problems	
	
Problem	Score	Problem	Score
Lack of regular physical activity.	11	Loneliness.	8
Existence of diagnosed chronic diseases.	11	Decreased self-esteem and self-perceived happiness.	8
Inadequate eating habits.	10	Decreased self-perceived visual and auditory acuity.	8
Low social interaction.	9	Housing conditions conducive to accidents.	7
Non-adherence to medicine regimen.	9	Poor awareness of parish support institutions for older adults.	7
Non-adherence to officially recommended influenza vaccination schedule.	9	Lack of motivation to engage in activities for personal development and future projects.	7
Lack of oral health care.	9	Lack of family doctor.	7
Self-perceived overweight condition.	9	Feeling insecure.	6
Occurrence of falls and fear of falling.	9	Inadequate attitude towards being a potential victim of violence.	6
		Cohabitation with smokers.	5

Stage 3 consisted in the ranking of the nine highest-scoring problems in the previous stage by experts in the area of gerontology and community nursing and public health, thus adding to the analysis their specialized knowledge about the topics being debated. That resulted in a reordering of the list of priority problems (Table 4). In step 4, the five problems most highly rated by the experts were analyzed in a systematic and organized way, according to the four components defined in the Hanlon method (Table 5). The application of those components – magnitude, seriousness, effectiveness, and feasibility (which includes the PEARL component, discussed with the community) resulted in the selection of the priority problem for intervention in the given community of older adults: low social interaction in community participation.

**Table 4:** Ranking of nine problems by experts. Fajã de Baixo, Portugal, 2012.

Problem	Expert 1	Expert 2	Expert 3	Average	Ranking
Non-adherence to medicine regimen	1	1	2	1.3	1
Low social interaction	2	6	1	3.0	2
Lack of regular physical activity	3	3	4	3.3	3
Occurrence of falls and fear of falling	5	2	3	3.3	4
Inadequate eating habits	4	5	5	4.7	5
Self-perceived overweight condition	6	4	6	5.3	6
Existence of diagnosed chronic diseases	7	8	7	7.3	7
Lack of oral health care	8	7	9	8.0	8
Non-adherence to influenza vaccination	9	9	8	8.7	9

**Table 5:** Final ranking of problems following use of the Hanlon Method. Fajã de Baixo, Portugal, 2012.

Problems	Size	Seriousness	Effectiveness	Feasibility	(A+B) C x D
			
	(A)	(B)	(C)	(D)	
Low social interaction	10	9	1	1	19
Lack of regular physical activity	10	7	1	1	17
Inadequate eating habits	10	8	1	1	18
Non-adherence to medicine regimen	10	10	0.5	1	10
Occurrence of falls and fear of falling	6	9	1	1	15

## DISCUSSION

This article described systematically the steps, strategies and procedures that enabled the selection of a priority problem from an initial set of 37 needs identified in a community health diagnosis.

Prioritizing means establishing a hierarchy of identified problems in order to determine priorities for intervention, i.e., classifying health problems according to their importance, effectiveness, and the feasibility of proposed interventions^10^.

Pallàs et al. (2003) point out that any prioritizing process involves subjectivity which cannot be completely eliminated due to several factors that influence the health dynamics of a community. Acknowledging the interference of subjectivity in these processes highlights the need to clarify strategies and steps in an orderly and systematic way to achieve a more transparent, well-grounded and auditable process^5^. The uniqueness of each prioritization process results in the incorporation of different “views” by various professionals. It also receives important contributions from sources outside community intervention teams, as well as from the community itself. In the prioritization process herein presented, different perspectives are identified at different moments of the process: 1) intervention team; 2) experts; 3) community.

## PERSPECTIVE OF THE INTERVENTION TEAM

This focused on the work of the members of the community intervention team. It comprised organizing the findings from the community health diagnosis, namely grouping the health problems/needs according to their level of similarity and classifying them by applying epidemiological criteria.

Analyzing reflexively the procedures used in the prioritization process, a first evaluation by the intervention team was considered relevant. The definition of a preliminary list of priority problems, supported by continuous literature review and processes of debate, consensus and minimization of subjectivity, ensured a better organization of the information, and thus a better-grounded and more efficient prioritization process. This strategy guaranteed that the issues presented to the experts and community for debate were centered on a restricted set of needs, avoiding dispersion and contributing to the focused participation of those stakeholders.

## PERSPECTIVE OF THE EXPERTS

In the next phase, the incorporation/intervention of external perspectives in analyzing the problems of the prioritization process was deemed important. Pallàs et al. (2003) reaffirm the importance, in setting priorities for problem analysis and decision-making, of incorporating contributions other than those of the members of the actual intervention teams^10^.

Of the 19 highest-scoring problems in the previous phase, nine were selected in the independent ranking of the experts. Their intervention made an important contribution to evaluating the problems by enabling a broad “external look” with different perspectives of the themes under debate.

The experts’ work revealed dissonance with the order previously established by the team, since only the lack of regular physical activity was classified by both experts and team among the three highest-scoring problems. On the other hand, in a broader analysis, the experts’ intervention allowed a clearer differentiation of the ordering of problems. The six problems classified by the team with the same score (ranked in fourth position – Table 3) were differentiated by the experts, who assigned them different positions (Table 4).

The joint process of problem selection and analysis by the team and experts enabled the identification and ordering of the five most pertinent interventions in terms of potential health gains.

The different analyses of the various professionals enriched the process of prioritizing and debating the problems encountered. They stressed the need for the community to be involved in the decision-making process regarding issues that affect them directly^6,15^.

## PERSPECTIVE OF THE COMMUNITY

One of the essential assumptions for the success of health interventions in communities is the importance of their playing an active role in identifying and minimizing existing or potential health problems, in order to promote higher levels of health and well-being. The concept of empowerment acquires special relevance. It is understood as a social process by which individuals, organizations and communities develop expertise in solving challenges in a context of action/change in the social and political environment, aimed at acquiring better levels of quality of life and equity^6,15^.

The Hanlon method was chosen to ensure the community’s involvement in the prioritization process. One of the requirements for its use is the analysis of problems and feasibility of interventions together with the community.

This method, which combines criteria of problem importance (magnitude and seriousness) and criteria of resolution capacity (solution effectiveness and intervention feasibility), complemented the analysis process, incorporating the community’s perspective on the propriety of action related to the various problems, and assessed their level of acceptability. The problems were contextualized in the environment in which they happen by those who experience their influence, assessing the existence of potential resources for community intervention. Using the focus group to incorporate the community’s input in setting priorities was one of the key steps in the process.

The focus group is an established social research technique organized as a structured discussion, involving the progressive sharing and clarification of participants’ points of view and ideas. It is particularly interesting for analyzing topics that may give rise to different opinions or bring up complex issues that need to be explored with greater precision^3^.

The use of this technique allowed the sharing of several perspectives and experiences on the subjects under debate, enabling a clear assessment of the position of the various actors in relation to each problem.

The debate translated a process of mediation between the team and the community, stimulating reflection on the issues being discussed. This contributed to deciding on the priority problem and developing critical reasoning and capacity for future intervention.

The prioritization process, with the ensuing selection of the priority problem, led to the implementation of a community intervention project, developed with the community of older adults. The objective was to increase their levels of community participation and strengthen social interaction.

Health planning is a demanding and meticulous development process. Its implementation is essential for community work. Health diagnosis and the various existing prioritization methods are instruments available to health professionals who work at the community level and intend to engage in pertinent, multicausal issues.

The priority setting methodology implies the development of a framework for the construction of a process based on rigorous criteria, adaptable by each team and consensual for the different elements, in which community participation is mandatory. In the example herein presented, the use of different stages and, in particular, the Hanlon method, was an added value for the diversity of components and the expectation of community participation in decision making.

Starting out from a list of health problems identified by the performance of a health diagnosis in a community of older adults, and the definition of a multi-stage priority setting process that integrated different perspectives, it was possible to progressively select the identified problems. This culminated in the selection of the problem acknowledged as top priority.

This process, by including the different actors involved, is time consuming and implies the development of skills in managing and integrating different perspectives in multiple processes of discussion and negotiation. However, the integrated inclusion of the perspective of different actors (health professionals and community) allows a progressive analysis of the problems identified in a community. Thus, it leads to effective decision-making processes and adds community knowledge to the inflexibility of numbers. This balance may be the key to success in community health interventions, since it ensures that the problems identified are effectively valued by the communities.

The publication of more concrete experiences, describing how community health teams implement inclusive strategies to allocate scarce resources according to existing data, will be of great value to community and collective health professionals.

## References

[1] Buss PM, Pellegrini A (2007). A saúde e seus determinantes sociais. Physis.

[2] Chao R, Alvarez M (1996). Diagnóstico comunitário de la situation de salud. Rev Cubana Med Gen Integr.

[3] Geoffrion P, Gauthier B (2003). O grupo de discussão. Investigação social: da problemática à colheita de dados.

[4] Gonçalves A (2006). Problema de saúde pública: caraterizando e avaliando aplicações. Rev Bras Epidemiol.

[5] Keller L, Strohshein S, Briske L, Stanhope M, Lancaster J (2011). Prática de enfermagem de saúde pública centrada na população: a roda de intervenção. Enfermagem de saúde pública.

[6] Laverack G (2008). Promoção de saúde: poder e empoderamento.

[7] Lock S, Stanhope M, Lancaster J (2011). Prática baseada na evidência. Enfermagem de saúde pública.

[8] Martínez D, Palacio J, Oliver A, Fuente N, Mariscal C, Corral M (2007). Conceptos generales en atención primária para enfermaria.

[9] Pallàs J, Baiges J, Zurro A, Pérez J (2003). Análisis de la situación de salud. Atención primaria: conceptos, organización y práctica clínica.

[10] Pallàs J, Bertrán E, Baiges J, Zurro A, Pérez J (2003). Bases para la programación en atencíon primaria. Atención primaria: conceptos, organización y práctica clínica.

[11] Paúl C (2005). Envelhecimento activo e redes de suporte social. Rev Fac Letras Univ Porto: Sociologia.

[12] Preteleiro M, Marques R, Galhardo T (2004). Uma análise crítica das orientações estratégicas: o plano nacional de saúde português.

[13] Rodrigues R, Crespo S, Loureiro L, Silva S, Azeredo Z, Silva C (2015). Os muito idosos: avaliação funcional multidimensional. Rev Enferm Refer.

[14] Veras R (2009). Envelhecimento populacional contemporâneo: demandas, desafios e inovações. Rev Saude Publica.

[15] Wiggins N (2011). Popular education for health promotion and community empowerment: a review of the literature. Health Promot Int.

[16] World Health Organization (2002). Active ageing: a policy framework.

